# 

**Published:** 1874-12

**Authors:** 


DECEMBER, 1874.

### THE AMERICAN JOURNAL OF DENTAL SCIENCE,

EDITED BY

F. J. S. GORGAS, M. D., D. D. S.

VOL. 8.—THIRD SERIES.—No. 8.

BALTIMORE:

SNOWDEN & COWMAN.

LONDON:

TRUBNER & CO., 60 PATERNOSTER ROW.

1874.

PAYABLE IN ADVANCE.

PUBLISHED MONTHLY.

$2.50 PER ANNUM.

